# DNA methylation in interleukin-11 predicts clinical response to antidepressants in GENDEP

**DOI:** 10.1038/tp.2013.73

**Published:** 2013-09-03

**Authors:** T R Powell, R G Smith, S Hackinger, L C Schalkwyk, R Uher, P McGuffin, J Mill, K E Tansey

**Affiliations:** 1 King's College London, MRC Social Genetic and Developmental Psychiatry (SGDP) Centre, Institute of Psychiatry, London, UK; 2 Brain Repair Centre, Dalhousie University, Halifax, NS, Canada; 3 University of Exeter Medical School, University of Exeter, Exeter, UK

**Keywords:** antidepressants, biomarker, cytokines, epigenetics, pharmaco-epigenetics, pharmacogenetics

## Abstract

Transcriptional differences in interleukin-11 (*IL11*) after antidepressant treatment have been found to correspond to clinical response in major depressive disorder (MDD) patients. Expression differences were partly mediated by a single-nucleotide polymorphism (rs1126757), identified as a predictor of antidepressant response as part of a genome-wide association study. Here we attempt to identify whether DNA methylation, another baseline factor known to affect transcription factor binding, might also predict antidepressant response, using samples collected from the Genome-based Therapeutic Drugs for Depression project (GENDEP). DNA samples from 113 MDD individuals from the GENDEP project, who were treated with either escitalopram (*n*=80) or nortriptyline (*n*=33) for 12 weeks, were randomly selected. Percentage change in Montgomery–Åsberg Depression Rating Scale scores between baseline and week 12 were utilized as our measure of antidepressant response. The Sequenom EpiTYPER platform was used to assess DNA methylation across the only CpG island located in the *IL11* gene. Regression analyses were then used to explore the relationship between CpG unit methylation and antidepressant response. We identified a CpG unit predictor of general antidepressant response, a drug by CpG unit interaction predictor of response, and a CpG unit by rs1126757 interaction predictor of antidepressant response. The current study is the first to investigate the potential utility of pharmaco-epigenetic biomarkers for the prediction of antidepressant response. Our results suggest that DNA methylation in *IL11* might be useful in identifying those patients likely to respond to antidepressants, and if so, the best drug suited to each individual.

## Introduction

Major depressive disorder (MDD) is predicted to be the second leading cause of disability by 2020.^1^ Antidepressants are currently the first line of treatment for MDD, but around two-thirds of patients fail to respond to the first antidepressant prescribed, and a third fail to respond to multiple antidepressant treatments.^2^ Studies have attempted to establish biomarkers to predict response to antidepressant medication and to personalize treatment. Genetic biomarkers have been investigated as predictors of clinical outcome; however, results from large-scale pharmacogenetic studies have mostly been unsuccessful in identifying genes that are robustly associated with clinical antidepressant response.^3, 4, 5, 6, 7^

However, recent evidence draws further support to results from one genome-wide association study performed in the Genome-based Therapeutic Drugs for Depression project (GENDEP), which identified a single-nucleotide polymorphism (SNP) (rs1126757) in interleukin-11 (*IL11*) that predicted response to the selective serotonin reuptake inhibitor escitalopram.^4^ Further investigation of *IL11* at the transcriptional level found it to be expressed at a lower level in responders compared with that in non-responders after treatment with escitalopram, but not before the initiation of escitalopram treatment.^8^ Gene expression differences after treatment were partially mediated by rs1126757, implicating rs1126757 as a treatment-emergent expression quantitative trait locus.^8^

Similarly to expression quantitative trait loci, DNA methylation can also affect transcription factor binding and moderate gene expression changes, and DNA methylation has been linked to the pathophysiology of mood disorders.^9, 10^ Subsequently, in this study we aimed to investigate whether baseline DNA methylation in *IL11* could be used to predict antidepressant response.

The current study used blood samples collected from 113 individuals diagnosed with MDD as part of the GENDEP project. We attempted to identify: (1) whether there are DNA methylation predictors of general antidepressant response (independent of drug or genotype); (2) whether there are differential DNA methylation predictors, which could be used to predict whether an individual is more likely to respond to the antidepressant escitalopram or nortriptyline; and (3) whether there is an interaction between rs1126757 genotype and DNA methylation, which predicts response to antidepressants.

## Materials and methods

### Clinical sample

Patient samples were taken from the GENDEP project, which has been described in detail elsewhere.^11^ Briefly, GENDEP is a 12-week, partially randomized, open-label pharmacogenetic study with two active treatment arms. A total of 868 treatment-seeking adults (men: *n*=321; women: *n*=547) with MDD of at least moderate severity according to the ICD-10 or DSM-IV criteria were recruited from 9 European centers. Patients were aged 19–72 years and were of Caucasian European parentage. Diagnoses were established using the semi-structured Schedules for Clinical Assessment in Neuropsychiatry interview.^12^ Exclusion criteria were personal and family history of schizophrenia or bipolar disorder, current substance dependence, or whether participants had previously taken both of the drugs and demonstrated treatment resistance. Eligible participants were allocated to treatment with either the selective serotonin reuptake inhibitor escitalopram (total *n*=394) or the noradrenaline reuptake inhibitor nortriptyline (total *n*=312), which differed by antidepressant mechanisms of action.^13^ Patients with no contraindications were randomly allocated to a flexible-dosage of nortriptyline (50–150 mg daily) or escitalopram (10–30 mg daily) for 12 weeks. Patients with contraindications for one drug were offered the other. The GENDEP project was approved by ethics boards of participating centers, and all participants provided a written consent after the procedures were explained. GENDEP is registered at EudraCT (No.2004-001723-38, https://eudract.ema.europa.eu/) and ISRCTN (No. 03693000, http://www.controlled-trials.com).

Participants were assessed for severity of depressive symptoms by using the clinician-rated Montgomery-Åsberg Depression Rating Scale (MADRS) on a weekly basis.^14^ Previous work found that the MADRS scores were the most sensitive, clinically representative and internally consistent scores for depression symptom changes in GENDEP, and, consequently, is the measure we use in this study.^15^

A subset of 113 individuals (males *n*=46; females *n*=67; age 40.3 years±11.7 years) were randomly selected among patient samples, who had complete clinical data for the full 12 weeks of treatment and had genome-wide association study genotype data. All patients had a diagnosis of moderate to severe MDD, with a baseline severity of 27.7±4.8 (average MADRS score, s.d.). Individuals were treated with either the antidepressant escitalopram (*n*=80) or nortriptyline (*n*=33). Fewer than 10% of individuals had previously taken an antidepressant on entering GENDEP and all individuals were drug-free for 2 weeks before the start of the study. Before this, patients had reported taking antidepressants (*n*=10), benzodiazepines (*n*=35) and hypnotics (*n*=14). The average duration of the current depressive episode in our sample was 21.3±19.3 weeks (average duration, s.d.). Seventy-two of our patients had experienced a stressful life event within 6 months before entering the GENDEP study, as measured using the List of Threatening Experiences Questionnaire.^16^ Percentage change in the MADRS score from baseline to week 12 was used as a measure of antidepressant response. Higher positive changes in the percentage MADRS represent better treatment response.

### Experimental details

#### Genotyping

Patient blood samples were collected and stored in ethylenediaminetetraacetic acid (EDTA), after which DNA was extracted using a standard extraction procedure.^17^ Genotype data were collected as part of a genome-wide association study.^4^ Full details of genotyping and quality-control measures can be found elsewhere.^4^ Briefly, samples were sent to the Centre National de Genotypage (Evry, France) and were genotyped using the Illumina Human 610-Quad BeadChips (Illumina, San Diego, CA, USA), which genotypes more than 600 000 SNPs. All 113 patients included in the current study passed the routine quality-control tests, which included removing individuals for ambiguous sex, abnormal heterozygosity, cryptic relatedness (up to third-degree relatives), genotyping incompleteness (<97% coverage) and non-white European admixture. Genotype data include the rs1126757 SNP. Genotype data for this SNP was extracted using PLINK.^18^

#### DNA methylation

All DNA samples were quantified and tested for purity using the Nanodrop ND1000 (Thermo Scientific, Wilmington, DE, USA). Previous quality-control measures were employed for the purpose of genotyping and DNA had been stored at −80 °C.

Four hundred nanograms of genomic DNA was treated with sodium bisulfite using the EZ-96 DNA Methylation Kit (Zymo Research, Irvine, CA, USA) following the standard manufacturer's protocol. *IL11* primer design was based on *in-silico* bisulfite-amplicon prediction using the Mass array package (Bioconductor, www.bioconductor.org) in R (http://www.R-project.org). Primers were designed to span the CpG island in *IL11* (chr 19: 55880511–55880989, Genome Reference Consortium GRCh37/USCS version hg19, University of California Santa Cruz Genome Browser). Forward primers consisted of the following sequence: 5′-GATGGAGTTGGAGGTTTTAAGTTTTA-3′. Reverse primers consisted of the following sequence 5′-ACCCATAACTCTACCCCTCTCC-3′. For each 10 μl reaction, the polymerase chain reaction mastermix consisted of the following: 1 μl 10 × buffer (Qiagen, Crawley, UK), 0.2 μl dNTPs (10 μM; Thermo Scientific, Northumberland, UK), 0.2 μl MgCl_2_ (Thermo Scientific, UK), 0.1 μl HotStarTaq Polymerase (Qiagen), 1 μl *IL11* forward primer (Sigma-Aldrich, Poole, UK), 1 μl *IL11* reverse primer (Sigma-Aldrich), 2 μl DNA and 4.5 μl water. Thermal cycling conditions consisted of an initial enzyme activation stage (95 °C for 10 min); followed by 35 cycles of denaturation (95 °C for 30 s), hybridization (58 °C for 30 s) and extension (72 °C for 1 min); and a final single-extension step (72 °C for 4 min) and cool-down step (4 °C for 10 min).

Controls included both artificially hypermethylated DNA (positive control) and RNAse-free water (no template control). Polymerase chain reactions were performed in duplicate and the products were pooled together to reduce technical variation. DNA methylation was quantitatively assessed using the Sequenom EpiTYPER system (Sequenom, San Diego, CA, USA) as described previously.^19^ Data generated from the EpiTYPER software were filtered using in-built quality-control parameters, and CpG units with low call rates (that is, <90% call rates) were removed.

### Statistical analysis

Statistical analyses (i–iii) included age, sex, center of recruitment, baseline MADRS score and allocated antidepressant drug as covariates. For CpG units displaying non-normal distributions, we applied the square-root transformation.

#### (i) DNA methylation as a predictor of general antidepressant response

To investigate whether DNA methylation in *IL11* could be used as a predictor of general antidepressant response, we performed univariate linear regressions with percentage MADRS change as the dependent variable and CpG unit methylation included as a covariate.

#### (ii) Differential drug by DNA methylation predictors of antidepressant response

To assess whether CpG unit methylation might interact with our two antidepressant drugs to differentially predict an antidepressant response, we performed univariate linear regressions. Percentage MADRS change was selected as our dependent variable and covariates included CpG unit methylation and the interaction between the allocated antidepressant drug and the CpG unit methylation.

#### (iii) DNA methylation × rs1126757 predictors of antidepressant response

To investigate whether there was an interaction between rs1126757 and CpG unit methylation that could predict antidepressant response, we performed a univariate linear regression with percentage MADRS change as the dependent variable, and covariates including rs1126757 genotype, CpG unit methylation and the interaction between the CpG unit methylation and rs1126757.

#### (iv) Multiple testing correction

We entered all *P*-values generated from analyses i–iii into a single false discovery rate calculation to generate *q*-values. We achieved this using an online false discovery rate web-based tool available at http://www.sdmproject.com/utilities/?show=FDR. All *q*-values ⩽0.1 were considered to be true effects.

#### (v) Effects of medication, episode duration and recent stressful life events on DNA methylation

To assess whether previous medication use, the duration of the current depressive episode or the presence of a recent stressful life event might be driving any of our false discovery rate-significant predictors, we performed secondary analyses. We performed a univariate linear regression with CpG unit methylation as the dependent variable, and use of benzodiazepine, antidepressants, hypnotics and the presence or absence of a recent stressful life event were included as binary covariates, with episode duration (weeks) included as a continuous covariate.

## Results

**Figure 1:**
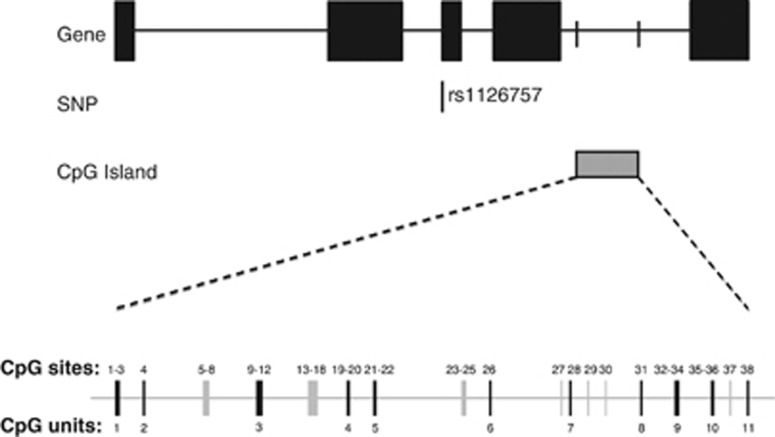
Schematic diagram of interleukin-11 (*IL11*) in a 5′ to 3′ direction, with the grey box showing the CpG island our assay covers and black boxes representing exons (top). Pictogram representing the individual CpG units within the CpG island, with black lines noting the CpG units adequately detected by the Sequenom and grey lines showing CpG units not assessed by this method (bottom).

**Figure 2:**
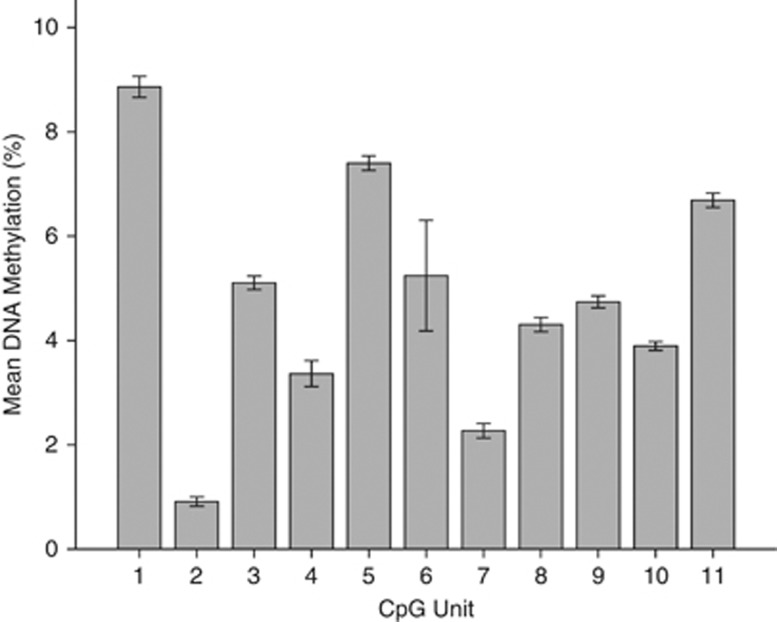
Bar graph showing mean percentage DNA methylation in our total sample at each of the 11 CpG units spanning the interleukin-11 (IL11) CpG island. CpG unit location is shown on the *x*-axis and methylation (%) is shown on the *y*-axis.

Results from DNA methylation experiments revealed that all positive controls (hypermethylated DNA) showed greater than 85% detected levels of methylation and all no template controls (H_2_O) showed 0% methylation. Eleven out of a possible 18 CpG units were adequately detected by the Sequenom and passed quality-control steps (Figure 1). Mean levels of the CpG unit methylation (%) and standard error (s.e.) at each of the 11 CpG units can be seen in Figure 2.

### DNA methylation as a predictor of general antidepressant response

**Figure 3:**
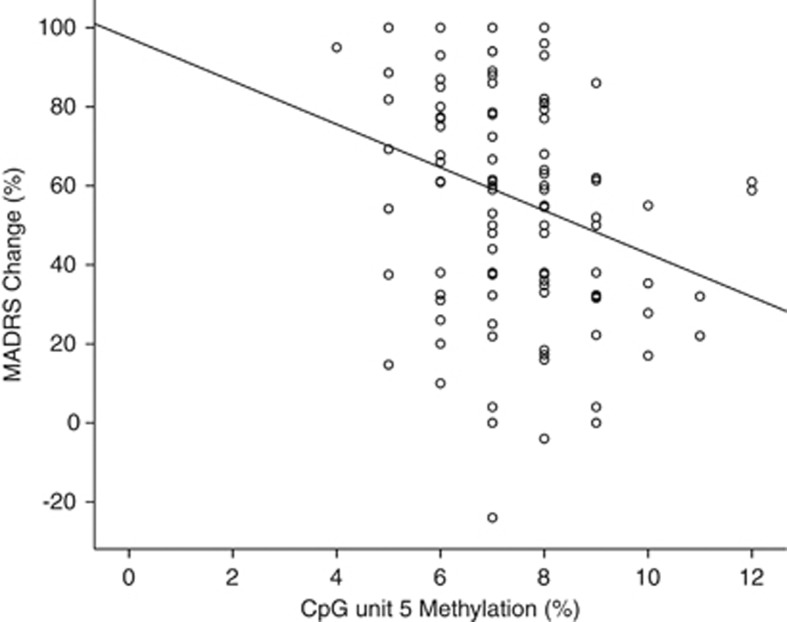
Scatter plot of the relationship between DNA methylation at CpG unit 5 (*x*-axis) and percentage Montgomery-Åsberg Depression Rating Scale (MADRS) change (*y*-axis). Line represents the line of best fit. DNA methylation at CpG unit 5 significantly predicted percentage MADRS change in our model (*P*=0.005).

A univariate linear regression was performed to assess whether methylation levels at CpG units in *IL11* predicted response to either antidepressant medication. CpG unit 5 significantly predicted antidepressant response (F=8.429, d.f.=1, *η*_p_^2^=0.082, *P*=0.005, *q*=0.055; Table 1). Lower levels of DNA methylation at CpG unit 5 was associated with better response to antidepressants (Figure 3).

### Differential DNA methylation by drug predictors of antidepressant response

**Figure 4:**
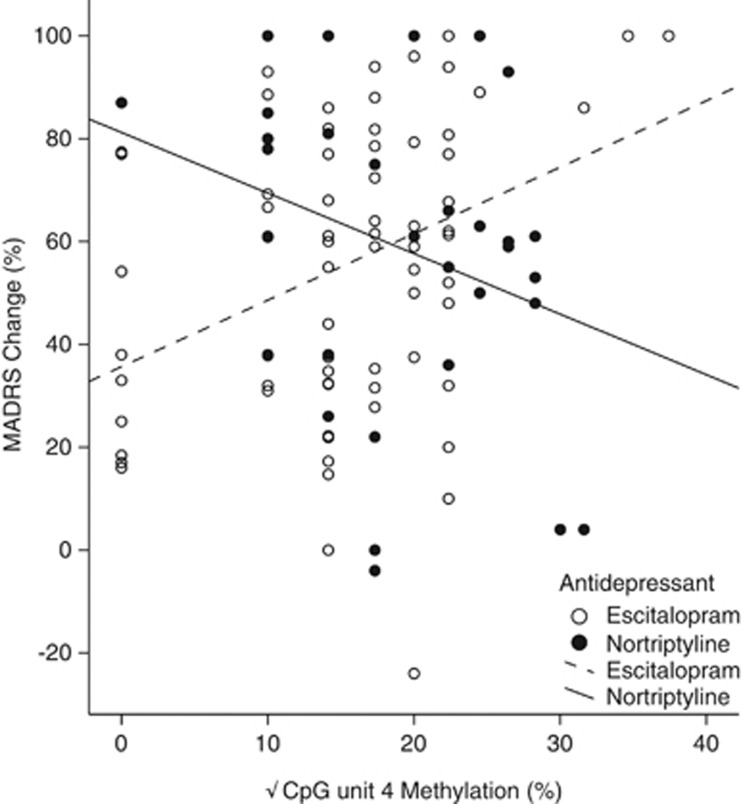
Scatter plot of the relationship between percentage DNA methylation at CpG unit 4 (*x*-axis) and percentage Montgomery-Åsberg Depression Rating Scale (MADRS) change (*y*-axis). Lines represent line of best fit for each drug group. DNA methylation at CpG unit 4 was found to significantly interact with the drug type to predict percentage MADRS change in our model (*P*=0.005).

Univariate linear regressions were performed to assess whether DNA methylation at any of the CpG units in *IL11* acted as a predictor of differential response. Methylation at CpG unit 4 significantly predicted differential response to treatment (F=8.412, d.f.=1, *η*_p_^2^=0.083, *P*=0.005, *q*=0.055; Table 1). Higher levels of DNA methylation at CpG unit 4 was associated with better response in individuals taking escitalopram, but was associated with worse response in those taking nortriptyline (Figure 4).

### DNA methylation by rs1126757 interaction predictors of antidepressant response

**Figure 5:**
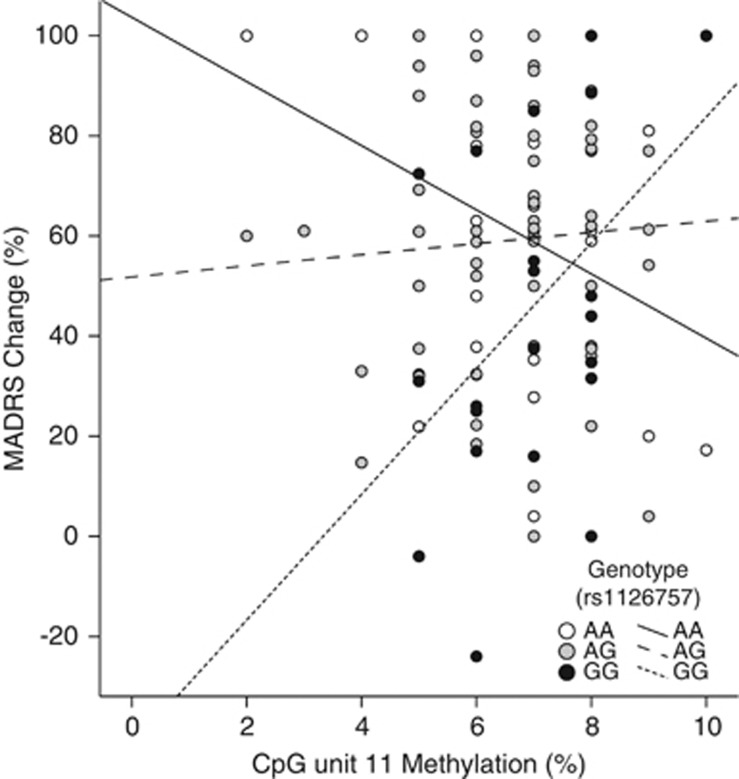
Scatter plot of the relationship between percentage DNA methylation at CpG unit 11 (*x*-axis) and percentage Montgomery-Åsberg Depression Rating Scale (MADRS) change (*y*-axis). Data points and lines of best fit correspond to different genotypes of the genome-wide association study single-nucleotide polymorphism, rs1126757. DNA methylation at CpG unit 11 was found to significantly interact with rs1126757 to predict percentage MADRS change in our model (*P*=0.002).

A univariate linear regression was performed to test whether an interaction between methylation at any CpG unit and rs1126757 genotype predicted antidepressant response. An interaction between methylation at CpG unit 11 and rs1126757 significantly predicted response to treatment (F=6.821, d.f.=2, *η*_p_^2^=0.131, *P*=0.002, *q*=0.055; Table 1). Individuals homozygous for the G-allele (GG), who had higher levels of methylation at CpG unit 11, responded better to antidepressant treatment than those individuals homozygous for the A allele (AA), with no effect of DNA methylation observed in heterozygotes (AG) (Figure 5).

### Testing for confounding factors

Linear regressions revealed that none of our possible confounding factors (previous medication use, duration of depressive episodes, or occurrence of a recent stressful life event) were driving DNA methylation in any of our three FDR-significant DNA methylation predictors.

## Discussion

MDD is becoming an increasing global concern, creating an urgent need for effective treatment.^1, 2^ With high interindividual variation in treatment response to antidepressants, the search for biomarkers aims to personalize therapy and improve upon our current ‘trial and error' method of treatment selection.^3^ Genetic, proteomic and, recently, transcriptomic biomarker studies have attempted to identify predictors of antidepressant response, with varied success.^3, 4, 5, 6, 7, 8, 20^ Our previous work identified a treatment-emergent expression quantitative trait locus (rs1126757) in *IL11*, which predicted response to the antidepressant escitalopram.^8^ The SNP driving the observed transcriptional differences had previously been identified as a predictor of response to escitalopram as part of a genome-wide association study.^4, 8^ Subsequently, we hypothesized that other baseline factors with the potential to affect transcription factor binding and gene expression changes in *IL11* might also predict antidepressant response. Here we explored the potential utility of DNA methylation in *IL11* as a baseline predictor of antidepressant response.

The results detailed here are the first to demonstrate the potential use of pharmaco-epigenetic biomarkers as the baseline predictors of antidepressant response. Our results suggest that CpG unit-specific DNA methylation in *IL11* could be used to predict whether an individual is likely to respond to antidepressants (see Figure 3) and, if they are, the type of drug best suited to each individual (see Figure 4). The interaction between genotype and DNA methylation also reveals the importance of integrating genotype and methylation data in search of molecular biomarkers for antidepressant response (see Figure 5).

IL11 has previously been found to induce potent inhibitory effects on serotonin signaling.^21^ Consequently, it adds to a growing number of functionally relevant cytokines, which have been previously associated with antidepressant response (for example, tumor necrosis factor and IL-6).^4, 8, 20, 22^ It also further supports suggestions that augmentation therapies targeting the cytokines might be useful in improving response to antidepressants.^23, 24, 25^

The cause of our observed DNA methylation differences remains unclear, but based on our results they are unlikely to be related to episode duration, current medication use or experiences of a recent stressful life event. In future studies, it would be interesting to test whether early stressful life events might predict DNA methylation in *IL11*, as early life stressors are known modulators of the methylome, affect the levels of inflammatory markers and act as a risk factor for MDD.^26, 27, 28, 29^

Despite the promising results detailed here, the study has three main limitations. First, the differences in DNA methylation observed are small and CpG unit-specific; hence, further studies are required to determine whether these differences are biologically meaningful. Second, although blood has useful biomarker properties (e.g., it is renewable, peripherally accessible and has access to the brain tissue), further work is still required to understand how DNA methylation differences in the blood might relate to the differences in the brain. Third, our method could not detect DNA methylation at all CpG sites, and in some cases it used averages across neighboring CpG sites to form CpG units (see Figure 1).

In conclusion, results presented here are the first to demonstrate the potential clinical utility of DNA methylation biomarkers as predictors of antidepressant response. Future studies are further needed to replicate these findings and validate the relationship between site-specific methylation in *IL11* and antidepressant response.

## Figures and Tables

**Table 1:** A summary of the results from the univariate linear regressions

	*DNA methylation*				*Drug × DNA methylation*				*Genotype × DNA methylation*			
*CpG unit*	*F*	*d.f*	P	q	*F*	*d.f*	P	q	*F*	*d.f*	P	q
1	0.002	1	0.965	0.966	0.097	1	0.757	0.961	0.784	2	0.459	0.854
2	0.784	1	0.378	0.854	1.672	1	0.199	0.854	0.742	2	0.479	0.854
3	0.058	1	0.810	0.966	0.051	1	0.821	0.966	0.080	2	0.924	0.966
4	1.438	1	0.234	0.854	8.412	1	**0.005**^a^	**0.055**	0.363	2	0.697	0.961
5	8.429	1	**0.005**^a^	**0.055**	2.477	1	0.119	0.854	0.135	2	0.874	0.966
6	0.853	1	0.358	0.854	0.109	1	0.742	0.961	0.034	2	0.966	0.966
7	0.327	1	0.569	0.854	0.327	1	0.569	0.854	0.711	2	0.494	0.854
8	0.407	1	0.525	0.854	0.407	1	0.525	0.854	1.911	2	0.154	0.854
9	0.566	1	0.454	0.854	1.533	1	0.219	0.854	0.594	2	0.554	0.854
10	0.525	1	0.470	0.854	0.525	1	0.470	0.854	0.756	2	0.472	0.854
11	0.010	1	0.920	0.966	0.131	1	0.718	0.961	6.821	2	**0.002**^a^	**0.055**

Results include an F statistic, d.f., *P*-values and *q*-values.

A summary of the results from the univariate linear regressions in which we tested whether (from left to right) DNA methylation, drug by DNA methylation interactions, or rs1126757 genotype by DNA methylation interactions in each of the 11 CpG units could predict antidepressant response.

a Significant *P*-values (*P*⩽0.005) are highlighted in bold.

## Notes

PM has received consultancy fees and honoraria for participating in expert panels from pharmaceutical companies, including Lundbeck and GlaxoSmithKline. All other authors declare no conflict of interest.
